# REGγ regulates circadian clock by modulating BMAL1 protein stability

**DOI:** 10.1038/s41420-021-00704-9

**Published:** 2021-11-05

**Authors:** Syeda Kubra, Haiyang Zhang, Youwen Si, Xiao Gao, Tianzhen Wang, Linian Pan, Lei Li, Nanzhe Zhong, Junjiang Fu, Bianhong Zhang, Xiaotao Li

**Affiliations:** 1 Shanghai Key Laboratory of Regulatory Biology, Institute of Biomedical Sciences, School of Life Sciences, East China Normal University, 500 Dongchuan Road, 200241 Shanghai, China; 2 Ministry of Education, Shanghai Key Laboratory of Brain Functional Genomics, East China Normal University, Shanghai, China; 3 Department of Orthopedic Oncology and Spine Tumor Center, Changzheng Hospital, Naval Medical University, 415 Fengyang Road, 200003 Shanghai, China; 4 Key Laboratory of Epigenetics and Oncology, The Research Center for Preclinical Medicine, Southwest Medical University, 646000 Luzhou, Sichuan China; 5 Department of Molecular and Cellular Biology, Dan L. Duncan Cancer Center, Baylor College of Medicine, One Baylor Plaza, Houston, TX 77030 USA

**Keywords:** Circadian regulation, Proteasome

## Abstract

Endogenous clocks generate rhythms in gene expression, which facilitates the organisms to cope through periodic environmental variations in accordance with 24-h light/dark time. A core question that needs to be elucidated is how such rhythms proliferate throughout the cells and regulate the dynamic physiology. In this study, we demonstrate the role of REGγ as a new regulator of circadian clock in mice, primary MEF, and SY5Y cells. Assessment of circadian conduct reveals a difference in circadian period, wheel mode, and the ability to acclimate the external light stimulus between WT and KO littermates. Compared to WT mice, REGγ KO mice attain the phase delay behavior upon light shock at early night. During the variation of 12/12 h light/dark (LD) exposure, levels of *Per1*, *Per2*, *Cry1*, *Clock*, *Bmal1*, and *Rorα* circadian genes in suprachiasmatic nucleus are significantly higher in REGγ KO than in WT mice, concomitant with remarkable changes in BMAL1 and PER2 proteins. In cultured cells depleted of REGγ, serum shock induces early response of the circadian genes *Per1* and *Per2* with the cyclic rhythm maintained. Mechanistic study indicates that REGγ directly degrades BMAL1 by the non-canonical proteasome pathway independent of ATP and ubiquitin. Silencing BMAL1 abrogates the changes in circadian genes in REGγ-deficient cells. However, inhibition of GSK-3β, a known promoter for degradation of BMAL1, exacerbates the action of REGγ depletion. In conclusion, our findings define REGγ as a new factor, which functions as a rheostat of circadian rhythms to mitigate the levels of *Per1* and *Per2* via proteasome-dependent degradation of BMAL1.

## Introduction

Earth’s atmosphere has the evolutionarily fundamental characteristic of 24-h light/dark (LD) cycle that effectuate the predominant influence on sleep–wake and activity of organisms. To coexist with the ambient variables such as light, dark, and temperature, organisms are created with a system of circadian clocks that work throughout the body, thus maintaining the life cycle upon changes in climate [1]. The mammalian circadian time keeping framework is primarily composed of the core circadian clock, the suprachiasmatic nucleus (SCN), a tiny pair of neuron clusters at the anterior hypothalamus and the subsidiary clocks in the most peripheral tissues [2]. SCN neurons instinctively operate the light signals received by the retina of eye and program the endogenous rhythms of body temperature, hormone secretion, feeding fasting, and locomotor behavior accordingly with external nature [3]. LD shifts are the major signals to orchestrate the SCN pacemaker, which subsequently work in coordination with peripheral clocks [4]. Exposure to transient light at night causes immediate changes in the circadian clock. It is predicted in the previous literature that light induces multiple signaling pathways to cause *Per1* and *Per2* gene expression and thus the phase changes in circadian rhythms [5]. Genetic perturbations of clock genes or environmental disturbances to circadian rhythms spark off various pathologies including sleep disorder, diabetes and cancer, suggesting that proper maintenance of circadian control is pivotal for vigorous health [6, 7].

At the molecular level, a core set of genes in cells in the way of transcriptional and post-translational feedback loops creates the molecular corpuscular circadian clocks, which coordinates with the metabolic cycles [8, 9]. The basic constituents of these molecular clocks include transcription factors CLOCK and BMAL1, which together forms heterodimers and binds through their PAS domain to the E-box elements of Period (*Per1*, *Per2*, *Per3*) and Cryptochrome (*Cry1*, Cry2) genes at the promoter regions. Induction of *Per* and *Cry* transcriptions ensures the performance of the positive core of the circadian clock. PER and CRY proteins translocate in the form of PER-CRY complex to the nucleus to obstruct the CLOCK-BMAL1-mediated transcriptional activity making the negative core of circadian clock. Therefore, the coordination functioning of these genes generates the ~24-h circadian biorhythms [10]. Orphan nuclear receptors REV-ERBα, REV-ERBβ, and RORα proteins work in another interconnecting feedback loop with CLOCK-BMAL1 and contribute to the transcriptional control of the *Bmal1* and *Clock* genes [11].

Circadian clock proteins undergo post-translational modifications (PTMs) to augment important pace in the molecular oscillations of circadian clocks. Post-translational processing of clock proteins is crucial to regulate significant biological functions such as intercellular localization of clock molecules and precise time keeping between active complex formation and repression of *Per* and *Cry* transcription. The circadian activator BMAL1 protein undergoes multitude of PTMs, including acetylation [12], phosphorylation and protein instability by kinases [13], sumoylation [14], and ubiquitylation [15]. BMAL1 is a unique core clock regulator and defects in BMAL1 leads to circadian disruption [16] and various abnormalities, such as defects in glucose–lipid metabolism [17, 18] early aging [19], skeletal mandibular hypoplasia [20], and cancer [21].

REGγ or PA28γ (encoded by *PSME3* gene) is a 28-kDa 11S proteasome activator. It was first discovered as a major autoantigen in the blood serum of patients with systemic lupus erythematosus [22]. REGγ has the intrinsic property that it binds and activates the proteasome to promote the ubiquitin- and ATP-independent cleavage of intact proteins [23, 24], mediating a degradation pathway distinguishable from the canonical ubiquitin–proteasome system [25]. Several important studies suggest that REGγ have important functions in cancer, immunity, and other pathophysiological processes [26–28]. However, the role of REGγ-20S proteasome in circadian rhythms has not been investigated.

In the current work, we have identified REGγ as a new regulator of circadian rhythms by downregulating the circadian genes via proteasome-dependent degradation of BMAL1. To illuminate the role of REGγ in circadian clocks, we used REGγ wild-type (WT) and knockout (KO) mice as well as REGγ-deficient mouse embryonic fibroblast (MEF), and SY5Y cells. REGγ KO mice achieved the phase delay behavior of running wheel upon light stimulus in constant dark/dark (D/D); however, the REGγ WT mice did not attain the circadian phase change phenomenon of wheel activity. We also found that REGγ KO mice had short free running period than the REGγ WT counterpart did. Analysis of circadian gene expression levels displayed that REGγ ablation promoted transcription of a number of key circadian clock genes including *Per1*, *Per2*, *Cry1*, *Clock*, *Bmal1*, and *Rorα* in REGγ KO SCN and in REGγ KO MEF and SY5Y ShR cells. Western blot analysis also indicated increased levels of BMAL1 and PER2 proteins in REGγ-deficient MEF and SY5Y cells, suggesting that REGγ impede the circadian genes and protein expression. Mechanistically, we found the degradation of BMAL1 by the REGγ–proteasome system in vivo and in vitro. Given the role of glycogen synthase kinase 3beta (GSK-3β), a ubiquitous serine-threonine kinase, in control of BMAL1 stability [13], inhibition of GSK-3β results in shortening of the circadian period in mammalian cells [29, 30]. Consistently, we found that downregulation of REGγ-GSK3β pathway increased the BMAL1 levels and elevated the circadian expression of circadian clock genes in MEF and SY5Y cells. Hence, we established a potential role of REGγ in the regulation of circadian clocks by specifically promoting direct proteasomal degradation of BMAL1, thereby, altering the circadian gene expression. Our results suggest a novel mechanism of REGγ–proteasome system that is crucial for modulation of circadian rhythms.

## Results

### Mice with REGγ deficiency exhibit circadian phase change upon light stimulus

**Fig. 1:**
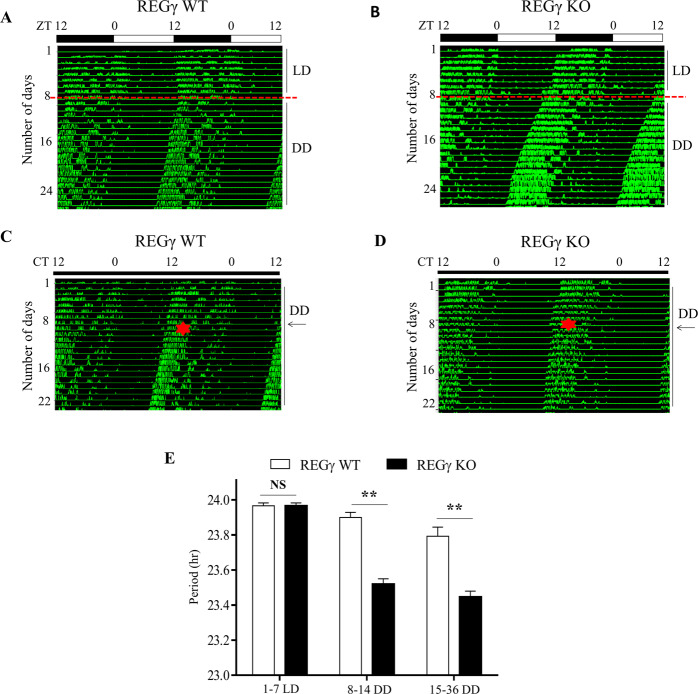
Light induced circadian phase shift in REGγ KO mice. **A** The actogram of 5-month-old REGγ WT control mice; **B** the actogram of littermate REGγ KO male mice in 12/12 h light–dark (LD) cycle followed by dark–dark (DD) for 3 weeks. For circadian phase change experiment, mice were first entrained to complete dark–dark (DD) cycle for 1 week and then placed in complete darkness for 3 weeks, and then a light pulse for 15 min at CT15 on day 8 in dark–dark (DD) was applied. REGγ WT mice in **C** did not accomplish the phase delay behavior of circadian rhythm, REGγ KO mice in **D** executed phase delay behavior upon light stimulus. Red dotted lines in **A**, **B** show the start day for constant darkness. Red star and black arrows in **C**, **D** indicate the day when a light pulse was applied. **E** Bar graph represent intrinsic periods (natural free running periods) of mice during the first week of 12/12 h LD cycle and initial 3 weeks in constant darkness. Values in **E** represent the mean ± SD. **p < 0.01; *t* test, WT vs. KO. The sample size was *n* = 5.

Circadian rhythms of physiology, behavior, and biochemical reactions are strongly synchronized by the endogenous circadian clocks. Wheel running activity is a relevant factor that alters the circadian rhythm of sleep–wake and feeding-fasting behavior of mice. Therefore, to study the association of REGγ with circadian clocks, we evaluated wheel running behavior of the mice. When acclimated to running wheels for 1 week in 12/12 h LD followed by 3 weeks of complete DD exposure, both WT and REGγ KO littermates exhibited free running rhythms in DD (Fig. 1A, B). Compared to WT mice, REGγ KO mice displayed short circadian period with less intermittent running wheel behavior (Fig. 1A, B). It is previously reported that transient light exposure causes resetting of the internal clocks and thus the locomotor activity of the animal [31]; thus we conferred the mice with a light pulse for 15 min at CT15 in the early night on day 8 of consecutive DD and then turned back to DD upon the completion of light pulse. While the light-induced circadian phase change had only minor effect in REGγ WT mice by actogram analysis (Figs. 1C and S1A), light pulse induced a significant circadian phase delay in the onset of running wheel activity in REGγ KO mice (Figs. 1D and S1B). The intrinsic periods were calculated based on the data reflecting wheel activity of the mice during the DD cycle. Intrinsic period (free running natural circadian period) of REGγ KO mice was ~23.5 h, while REGγ WT mice had a slightly longer period of 23.9 h, which was proximate to the previously published records for C57BL/6 mice [32] (Fig. 1E). The free running period of the REGγ KO mice was ~0.4 h shorter than that of REGγ WT mice. The results indicate that REGγ depletion contributes to the photic phase resetting of circadian behavior and modulates the circadian period in mice.

### REGγ ablation promotes transcription of circadian clock-specific genes in SCN

**Fig. 2:**
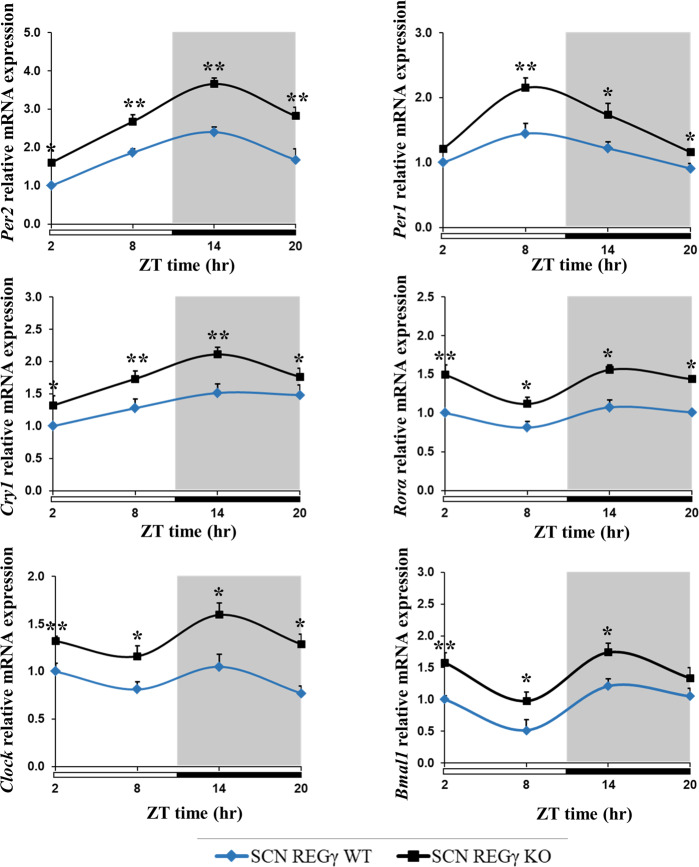
REGγ deficiency promotes the expression of circadian clock-specific genes in SCN. Real-time qPCR analysis of clock-specific genes including *Per1*, *Per2*, *Cry1*, *Clock*, *Bmal1*, and *Rorα* in REGγ WT and KO mice SCN. REGγ WT and REGγ KO mice were entrained to 12/12 h LD cycle, then sacrificed the mice at 6 h intervals on the last day of the cycle and the anterior hypothalamus tissues that contain the SCN were collected for mRNA analysis. Values represent the mean ± SD. **p < 0.01, *p < 0.05; *t* test, WT vs. KO.

SCN is the fundamental architect of circadian clock in mammals and the clock gene expression is essentially required by SCN so as to synchronize the peripheral tissues in phase with external environmental changes, thus SCN reserves the circadian time-keeping function [33]. Therefore, we tested whether REGγ deficiency may influence the expression of core circadian clock genes in mouse SCN. Real-time quantitative polymerase chain reaction (qPCR) and gel-based PCR analysis revealed that the basal mRNA expressions of circadian clock genes were significantly higher in SCN of REGγ KO mice than in WT control (Fig. S2A, B). Next, having established that the circadian clock genes are significantly upregulated in the SCN of REGγ KO mice compared to REGγ WT mice SCN, we entrained WT and REGγ KO mice in 12/12 h LD cycle for 7 days, sacrificed the mice at 6 h intervals of ZT (zeitgeber time), and collected the SCN tissues within anterior hypothalamus sections. From real-time qPCR analysis, we found that, despite of the systematic increase of clock-specific genes such as *Per2*, *Per1*, *Cry1*, *Clock*, *Bmal1*, and *Rorα* in REGγ KO SCN, the circadian genes follow similar patterns over ZT both in REGγ KO and WT SCN (Fig. 2). In line with previous reports, *Per2* mRNA also reached highest levels around ZT14, both in WT and REGγ KO SCN [34]. These observations suggest that REGγ deficiency increases the expression of clock-specific genes in SCN of REGγ KO mice compared to WT control.

### REGγ deficiency upregulates the rhythm of circadian genes in primary MEF and SY5Y cell line

**Fig. 3:**
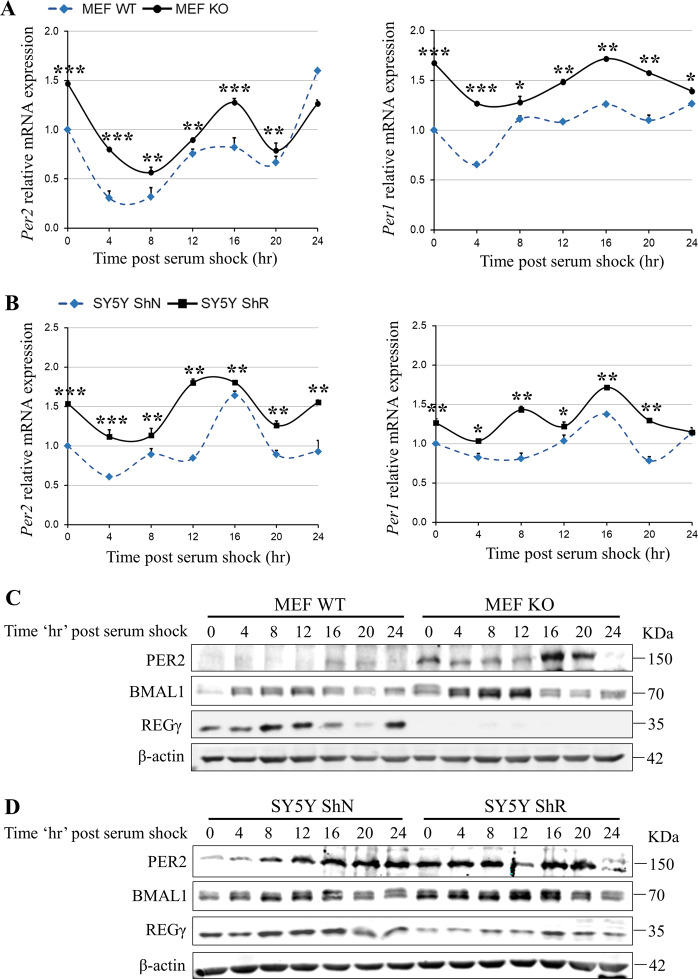
REGγ depletion affects the rhythms of circadian gene expression in cultured cell lines. Real-time qPCR analysis of circadian genes in **A** primary MEF WT/KO cells and **B** SY5Y ShN/ShR cells treated with 50% horse serum and then collected the cells at 4 h intervals across a 24-h cycle; the target gene expression levels were analyzed relative to the ribosomal RNA gene 18S. Immunoblots of REGγ and circadian components PER2 and BMAL1 in **C** MEF and **D** SY5Y cells. Values represent the mean ± SD. ***p < 0.001, **p < 0.01, *p < 0.05; *t* test, WT vs. KO; ShN vs. ShR.

To determine the influence of REGγ on rhythmic expression of the circadian genes in vitro, we conducted real-time qPCR and gel-based PCR analysis in REGγ WT/KO primary MEF and neuronal SY5Y cells with or without stable knockdown of REGγ (ShN and ShR cells). Consistent with in vivo expression of circadian genes in SCN, *Per1/2*, *Cry1*, *Clock*, *Bmal1*, and *Ror α* were expressed higher in MEF KO (Fig. S3A, B) and SY5Y ShR cells (REGγ knockdown cells) (Fig. S3C, D) compared to WT and ShN control cells. Given that the circadian oscillations are self-sustained in individual cell lines, so to validate the impact of REGγ on cyclic rhythm of circadian genes, we treated MEF and SY5Y cells with 50% horse serum for 2 h followed by serum-free medium and then harvested the cells at 4 h intervals for a 24 h cycle. Gel-based PCR analysis revealed that the rhythmic patterns of *Cry1*, *Clock*, and *Bmal1* genes in WT and REGγ-deficient MEF KO cells were generally indistinguishable (Fig. S4A); however, *Per2* and *Per1* mRNA were promptly induced with significant phase advance in response to serum shock in MEF KO cells compared to MEF WT cells (Figs. 3A and S4A). Similarly, the periodic levels of *Cry1*, *Clock*, and *Bmal1* transcripts in SY5Y ShR cells also had no profound differences to that in ShN cells (Fig. S4B). However, *Per2* and *Per1* mRNA were induced more readily in ShR cells compared to ShN cells (Figs. 3B and S4B). Consistent with changes in transcript levels, western blot results also indicated that both PER2 and BMAL1 protein expression levels were significantly elevated after serum shock in REGγ-depleted primary MEF KO (Fig. 3C) and SY5Y ShR (Fig. 3D) cells compared to control cells. These findings demonstrated that REGγ is critical for surveillance of normal circadian clock phenomenon in mammalian cells.

### REGγ negatively regulates BMAL1 by directly promoting its proteasome-dependent degradation

**Fig. 4:**
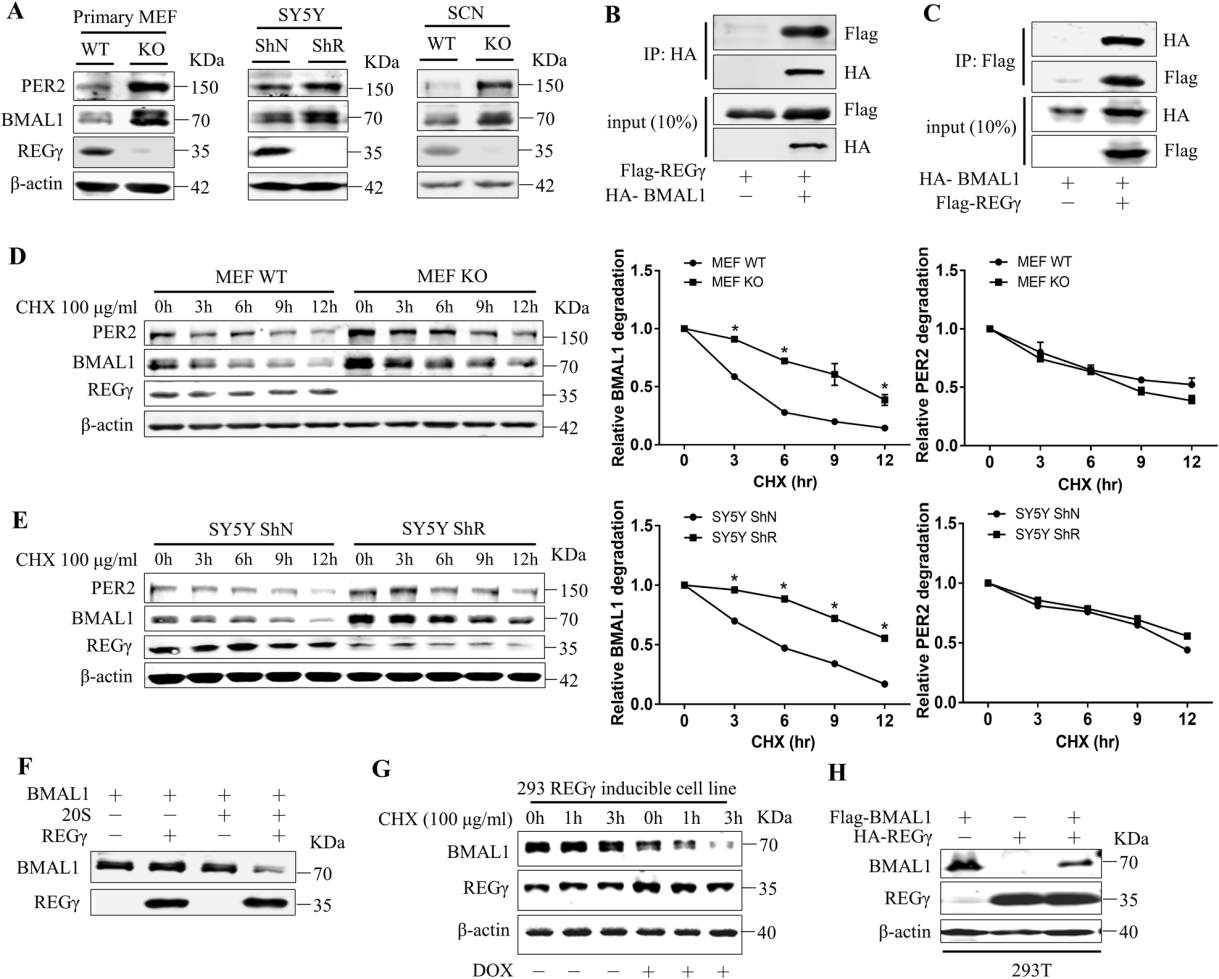
REGγ directly interacts with BMAL1 and promotes its degradation. **A** Expression of REGγ, BMAL1, and PER2 in MEF WT/KO, SY5Y ShN/ShR cells, and REGγ WT/KO mouse SCN. **B** Interaction between REGγ and BMAL1, determined by co-immunoprecipitation and western blot analysis following transient transfection of 2 μg of HA-BMAL1 and 2 μg of Flag-REGγ into 293T cells. **C** Reciprocal interaction between REGγ and BMAL1 was analyzed by transient transfection of 2 μg of Flag-REGγ and 2 μg of HA-BMAL1 into 293T cells. **D**, **E** Stability of endogenous BMAL1 in MEFs and in SY5Y cells was analyzed in the presence of CHX (100 μg/ml) for the indicated time points followed by western blot analysis. **F** In vitro proteolytic analysis of REGγ-mediated degradation of BMAL1. Purified REGγ, 20S proteasome, and in vitro-translated BMAL1 were incubated at 30 °C for 3 h as indicated. **G** Expression of REGγ was induced by doxycycline (1 μg/ml DOX for 48 h) in an engineered 293 cell line followed by western blot analysis of BMAL1 stability in the presence of cycloheximide as indicated. **H** 293T cells were transfected with Flag-BMAL1 and HA-REGγ followed by western blot analysis for REGγ and BMAL1 correlation. The quantitative results of BMAL1 stability in **D**, **E** were plotted to indicate dynamic changes. Values represent mean ± SD.  *p < 0.05; *t* test, WT vs. KO; ShN vs. ShR.

Analysis of PER2 and BMAL1 protein expression in cultured cells raised a question whether one of the key regulatory circadian proteins is targeted for degradation by the REGγ-20S proteasome pathway. Thus, we examined the overall expression of BMAL1 and PER2 proteins and we found that BMAL1 and PER2 protein were significantly high in REGγ KO mice SCN and MEF KO and REGγ knockdown SY5Y ShR than in WT controls (Fig. 4A). Given the role of BMAL1 as an upstream regulator of PER2, we tested whether BMAL1 is primarily targeted by the REGγ-20S proteasome pathway. Thus, physical interactions between REGγ and BMAL1 were analyzed in vitro, using exogenously expressed Flag-REGγ and HA-BMAL1 in 293T cells. Reciprocal co-immunoprecipitation suggested a robust interaction between the two proteins (Fig. 4B, C). We then investigated the dynamic stability of BMAL1 and PER2 proteins in the presence of cycloheximide (an inhibitor of de novo protein synthesis) for different times points in WT and REGγ KO MEF and SY5Y ShN/ShR cells. BMAL1 protein was degraded with slower rate in REGγ-deficient MEF and SY5Y ShR cells than in MEF WT (Fig. 4D) and SY5Y ShN control cells (Fig. 4E), which implies that REGγ may facilitate BMAL1 degradation. However, the decay rate for PER2 protein in WT and REGγ-depleted cells was comparable (Fig. 4D, E), suggesting that the negative regulation of PER2 by REGγ may be secondary to the changes in BMAL1 protein. To confirm whether BMAL1 is a direct target of the REGγ-20S proteasome pathway, we performed in vitro degradation assays with purified proteins in a cell-free system. Incubation of in vitro translated BMAL1 with 20S proteasome or REGγ alone could not facilitate the degradation of BMAL1. However, combination of REGγ and 20S proteasome promoted a significant proteolysis of BMAL1 in the absence of ATP (Fig. 4F). Next, we performed a gain-of-function experiment using a previously engineered REGγ-inducible 293 cell line in which a different allele of REGγ is expressed in the presence of doxycycline. Therefore, we carried out cycloheximide assay in doxycycline-treated cells and we found that the doxycycline-induced cells had an accelerated degradation of BMAL1 as compared to un-induced cells (Fig. 4G). 293T cells transfected with Flag-BMAL1 and HA-REGγ together also showed a decrease in the expression of BMAL1 protein (Fig. 4H). Together, these findings suggest that REGγ interacts directly with and promotes the degradation of BMAL1 protein, thus subsequently regulates the expression of downstream circadian genes.

### Alteration of circadian physiology induced by REGγ abrogation is GSK-3β-BMAL1 dependent

**Fig. 5:**
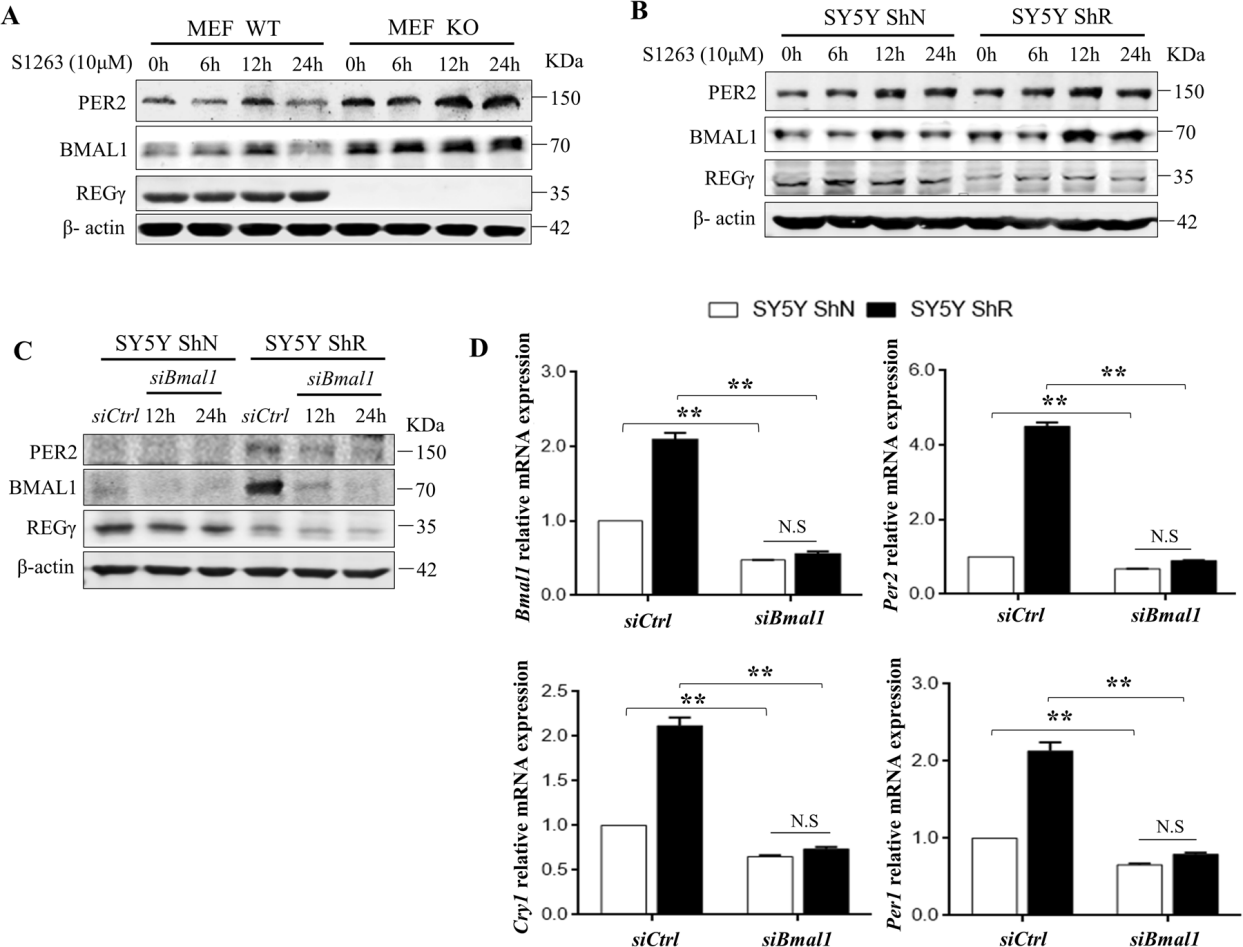
Alteration of circadian physiology induced by REGγ abrogation is GSK-3β-BMAL1 dependent. **A**, **B** MEF WT/KO and SY5Y ShN/ShR cells were treated with GSK-3β inhibitor (S1263, 10 μM) for the indicated time points followed by western blot analysis. **C** Expression of BMAL1 and PER2 levels following BMAL1 knockdown. **D** The mRNA expression of circadian genes *Per1*, *Per2*, *Cry1*, and *Bmal1* by real-time qPCR analysis in SY5Y ShN/ShR cells after the inhibition of *Bmal1* by si-RNA. The data were signified as mean ± SD. **p < 0.01; *t* test, si*Bmal1* vs. control, ShN vs. ShR.

Given that GSK-3β regulates BMAL1 protein stability and circadian function, we explored the regulation of circadian rhythms by REGγ following manipulation of BMAL1 protein levels in primary MEF and SY5Y cells. Indeed, treatment with GSK-3β inhibitor (S1263) significantly increased the expression of BMAL1 and PER2 protein levels and disrupted circadian patterns in both WT and REGγ KO MEF and SY5Y ShN and ShR cells compared to untreated cells (Fig. 5A, B). Similarly, upregulation of circadian genes including *Per1*, *Per2*, *Cry1*, *Clock*, and *Bmal1* was observed in cells treated with 50% horse serum shock in the presence of GSK-3β inhibitor (Fig. S4C, D), compared to control cells untreated with inhibitor (Fig. S4A, B). To substantiate the mechanism by which REGγ regulates circadian physiology via proteasomal degradation of BMAL1, we treated SY5Y cells with the small interfering RNA (siRNA) against *Bmal1* (si-*Bmal1*) and the control siRNA (si-Ctrl). Silencing *Bmal1* markedly decreased the expression of PER2 protein both in SY5Y ShN and ShR cells, attenuating the effect of BMAL1 and PER2 upregulation induced by REGγ knockdown (Fig. 5C). Consequently, ablation of *Bmal1* not only blocked the effect of REGγ depletion on circadian gene activation in ShR cells but also repressed overall expression of *Per1*, *Per2*, and *Cry1* genes in SY5Y ShN cells (Fig. 5D). These results indicate that REGγ inhibition is a previously unknown mechanism regulating circadian biology via controlling the homeostasis of BMAL1.

## Discussion

**Fig. 6:**
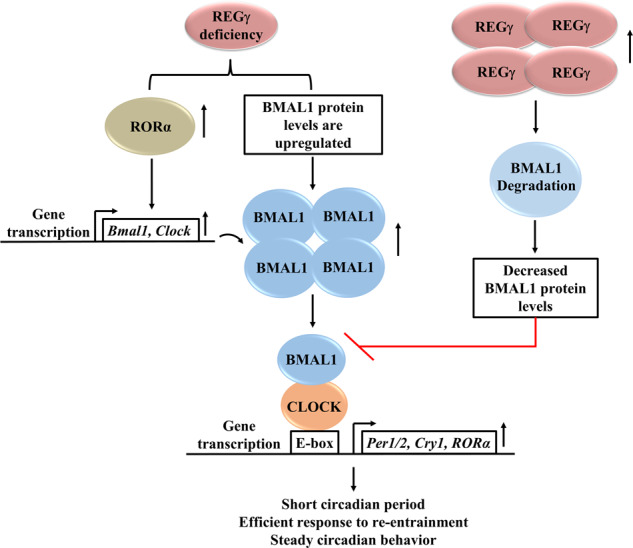
A Model of REGγ-dependent regulation of circadian rhythms. In mammalian cells, REGγ promotes rapid degradation of circadian transcription factor BMAL1 to attenuate the expression of circadian core genes, thus in mice, the downregulated circadian clock causes defect in circadian entrainment and disturbed circadian rhythms. However, mice having deficiency of REGγ display short circadian period, efficient response to re-entrainment, and steady circadian behavior.

Over recent years, research on REGγ has gained much emphasis due to its prominent functions in various biological pathways [35]. In the present work, we captivated the role of REGγ in circadian rhythms and proposed a mechanism by which REGγ impacts the expression of circadian genes in mice and in cell culture. We demonstrated that REGγ as a proteasome activator can specifically promote proteasomal degradation of the circadian protein BMAL1, but not the downstream PER2. In REGγ-deficient mice, the accumulation of BMAL1 accelerates the transcription of the circadian clock specific genes, such as *Per1*, *Per2*, *Cry1*, *Clock*, and *Rorα*, which may be related with the phenotype of circadian entrainment deficiency and disrupted circadian behavior (Fig. 6). Although several experiments including in vitro (cell-free system) have confirmed the direct degradation of BMAL1 by REGγ-20S proteasome. we have surprisingly found elevated level of mRNA transcript of *Bmal1* in REGγ KO mice SCN and in MEF KO and SY5Y ShR cells. Since *Rorα* was a target of CLOCK-BMAL1 and it contributes to the transcriptional activation of *Bmal1* and *Clock* genes [36], we observed that *Rorα* was upregulated in REGγ-depleted MEF and SY5Y cells and REGγ KO SCN, which may partially explain the increase of the mRNA level of *Bmal1* (Fig. 2). These results suggest a multi-layer regulation of BMAL1 by REGγ. From the analysis of the intrinsic periods by monitoring free running activity on running wheels, we found that the period of WT mice in DD cycle was 23.9 h, which was close to the values reported in other literatures [32, 37]. In contrast, REGγ KO mice had a short period of 23.5 h and showed consistent wheel activity rather than intermittent wheel activity, which was similar with that observed in SIRT1 overexpression mice [38], suggesting a blocking function of REGγ in circadian regulation.

Besides the master clock SCN, the circadian clock system also exists in almost all peripheral tissues such as liver, heart, lungs, and kidneys, which maintains circadian rhythm and regulates the expression of tissue-specific genes. It has been reported that SIRT1 exhibits distinct functions and mechanisms in SCN and peripheral tissues [38]. Therefore, we questioned the possibilities that the variation of REGγ expression over ZT in SCN or peripheral tissues could influence the expression levels of circadian genes. On the other hand, the abnormal REGγ expression/function may give rise to additional circadian rhythm-associated phenotypes. It will be interesting to see whether conditional KO and/or overexpression of REGγ in SCN vs. peripheral tissues may potentially lead to different phenotypes or functions via different mechanisms; all these needs to be verified by further experiments.

In recent years, more and more epidemiological and genetic data have shown that the disruption of circadian rhythms is linked to many disease-associated anomalies, such as depression, sleep disorders, cardiovascular disease, metabolic syndrome, and cancer. Metabolic rhythm disorder has been associated with multiple tumorigenesis and tumor development, including breast cancer, ovarian cancer, lung cancer, pancreatic cancer, prostate cancer, colorectal cancer, endometrial cancer, non-Hodgkin’s lymphoma, osteosarcoma, leukemia, head and neck squamous cell carcinoma, and liver cancer [39]. Tumor-suppressor genes, oncogenes, and circadian clock genes can be regulated by each other. For example, loss of circadian clock genes expression lead to the increased expression of oncogene c-Myc and causes metabolic disorder. While Myc could inhibit the expression of core clock genes [27, 40], the expression of p53 is circadian, and PER2 can directly regulate the activity of p53 [41]. It is previously found that, in SCN, p53 hinders the binding of CLOCK/BMAL1 complex with PER2 promoter, resulting in inhibition of *Per2* mRNA expression [42]. REGγ has been reported as an oncogene and play important roles in carcinogenesis and development of many kinds of cancers [43]. Whether and how REGγ and circadian clock genes reciprocally regulate each other in cancer progression deserve further exploration.

Transcriptional and post-translational events ensure the accuracy of circadian rhythms. SKP1, CULLIN1, F-box protein/β-TrCP ubiquitin ligase complexes have been reported to target PER and CRY proteins for degradation [44]. BMAL1 can be modified by hybrid E2/E3 enzyme UBE2O-mediated ubiquitination to modulate its stability and associated biological functions [45]. Our findings of REGγ-dependent action on circadian clock add an additional layer of regulation of the circadian system in vitro and in vivo, reinforcing the fine-tuned control of the key circadian elements. In conclusion, this study indicates that REGγ-20S proteasome acts via ubiquitin- and ATP-independent pathway to promote the degradation of BMAL1, adding a new insight of the regulatory mechanism in circadian protein degradation voyage.

## Materials and methods

### Mice

In this work, REGγ gene knock out mice and their WT littermate of C57BL/6 genetic inheritance were used, primitively provided by Professor John Monaco’s laboratory (College of Medicine, University of Cincinnati) [46]. Mice were kept in constant temperature of 21–22 °C and constant condition of 12/12 h LD cycle with access to food and water ad libitum. Mice were maintained according to ethical and scientific standards by “ECNU Multifunctional Platform for Innovation” (Grant Number: 011).

### Behavioral assay

Wireless mouse running wheel system (ENV-047) was used for recording of mice circadian rhythms. Five-month-old mice were randomly allocated to experimental groups without blinding. For assessment of the locomotor activity rhythms, REGγ KO and WT mice were housed in discretely ventilated running wheel cages for 2 weeks in constant 12/12 h LD sequence and then returned to complete darkness as a continuation of the dark phase of the last LD cycle. To examine the phase shift in locomotor activity, mice were exposed to light for 15 min at CT15 on day 8 in DD cycle and then returned to constant darkness for additional 8–10 days. The locomotor activities of mice and circadian phase differences were measured by the Med Associates Running Wheel Data Analysis Software. The sample size of our experiments was determined by our previous experience. The exact sample size was at least five.

### Cell culture and serum shock assay

Human SY5Y cell line obtained from Cell Bank of the Chinese Academy of Sciences, Shanghai, China, was cultured in Dulbecco’s modified Eagle’s medium (DMEM) F12 medium. HEK 293T, primary MEF, and REGγ-inducible 293 cell line were cultured in DMEM. All media were supplemented with 10% (vol/vol) fetal bovine serum (FBS) and 1% (vol/vol) (100 U/ml) HyClone penicillin–streptomycin (P/S) solution. Primary MEF cells were isolated from E13.5 day of REGγ HZ mouse embryos and REGγ-inducible 293 cell line was previously described [24]. Stable REGγ knockdown SY5Y cell line was generated by integration of retroviral ShREGγ vectors specific for REGγ to produce ShR (ShRNA against REGγ) or a control gene from OriGene (Rockville, MD) to produce ShN (ShRNA as a negative control) [47]. Cells were authenticated by a short tandem repeat profiling and routinely tested for mycoplasma contamination.

For serum shock assay, MEF and SY5Y cells were cultured in DMEM with 5% FBS and 1% PS. After confluence, at time = 0, the media was replaced with 50% horse serum (LOT# 1671371, GIBCO). Post 2 h of serum treatment, the high serum media was replaced with serum-free DMEM and collected the cells at 4 h intervals for protein and total RNA extracts as described in Aurelio Balsalobre procedure [48].

### Plasmids constructs and RNA interference

pcDNA3.1-flag-REGγ plasmid was constructed previously [49]. The REGγ ShR and ShN plasmids for stable knockdown of REGγ were previously described [28]. Full-length BMAL1 plasmid was constructed based on the sequence of human BMAL1 gene. A primer pair was designed to amplify the complete coding region of human BMAL1 gene and conducted PCR amplification in a final reaction volume of 25 μl, with 2 μl of cDNA from 293 T cells, 2 μl of primer mix (10 μM), and 12.5 μl of Premix Taq, then finally cloned into pcDNA3.1-HA vector.

siRNA targeted for *Bmal1* (F-5′-CCACCAACCCAUACACAGAAGCAAA-3′) and *GSK-3β* (F-5′-GCUCCAGAUCAUGAGAAAGCUAGAU-3′) were synthesized by Genepharma. SY5Y ShN/ShR cells were seeded in six-well plates and then transfected with the indicated amounts of siRNA with a Lipofectamine RNAiMAX Transfection Reagent Kit (Invitrogen,13778–075). Post 6 h of the siRNA transfection, the media was changed with DMEM containing 10% FBS, in the absence of P/S. The cells were incubated for 36 h, and the silencing efficiency was evaluated by western blotting and PCR analysis.

### Antibodies

Given are the primary and secondary antibodies used in western blot analysis with dilution ratios respectively. Anti-Period2-rabbit 1:500 (Abcam, ab180655), anti-BMAL1-rabbit 1:500 (Proteintech, 14268–1-AP), anti-REGγ-rabbit 1:250 (Invitrogen, 38–3900), anti-β-actin-mouse 1:1000 (Sigma, A5316), anti-flag-rabbit 1:1000 (MBL life science, M185–3L), and anti-HA-mouse 1:1000 (Abcam, ab130275). The fluorescent-labeled secondary antibodies, anti-Rabbit 1:5000 (IR800) specific to PER2, BMAL1, REGγ and Flag antibodies and anti-Mouse 1:10000 (M680) specific to β-actin and HA antibodies, were purchased from Jackson ImmunoResearch Laboratories, INC.

### Western blot analysis

Total protein from cultured cells and brain tissues were collected in cold RIPA lysis buffer having (50 mM Tris-HCl pH 7.5, 150 mM NaCl, 1% sodium deoxycholate, 1% Triton X-100, 0.1% sodium dodecyl sulfate (SDS), 5 mM EDTA, 1 mM Na3VO4, 5–10 mM NaF, 1 mM phenylmethanesulfonylfluoride (PMSF), and protease inhibitor cocktail (Roche)). Total protein concentrations were determined by the BCA Assay Kit (Green-to-purple, Beyotime, China) at 562 nm absorbance. Equal amounts of whole proteins (20 μg) were separated by 10% SDS–polyacrylamide gel electrophoresis (PAGE) gel along with molecular weight protein marker (PageRuler™ Prestained Protein Ladder, 10–180 kDa, ThermoFisher Scientific) and transferred the protein from the gel onto nitrocellulose membrane (Millipore, MA, USA). The membrane was blocked with 5% bovine serum albumin and then immunoblotted with primary antibodies at 4 °C overnight. After washing with PBST, the membrane was incubated with secondary fluorescent antibodies for 1 h and the specific signals of the immune-positive protein bands were observed by a fluorescent western blot infrared imaging system LI-COR Odyssey.

### Immunoprecipitation

HEK 293T cells were transiently transfected with specific amounts of pcDNA3.1-Flag-REGγ and pcDNA3.1-HA-BMAL1 plasmids (adjusted DNA 1000 ng with pcDNA3.1 vector) for 48 h. The cells were washed with ice-cold phosphate-buffered saline, collected in ice-cold EP tubes, and harvested for 40 min in IP lysis buffer (50 mM Tris-HCl pH: 7.5, 1 M EDTA, 150 mM NaCl, 10% Glycerol, 1% Nonidet P-40, protease inhibitor Cocktail, and PMSF). The lysates were incubated with HA or Flag beads (Sigma-Aldrich, Buchs, Switzerland) at 4 °C overnight. Washing procedures were applied on the complexes, denatured with 5× SDS protein loading buffer, and then finally boiled at 100 °C temperature. The samples were run on 10% SDS-PAGE gel to identify the protein–protein interactions.

### In vitro degradation assay

For in vitro degradation assay, REGγ protein was extracted and purified as described [23]. BMAL1 protein was translated using 50 µl in vitro translation system (TNT 40 µl, Milli-Q H2O 7 µl, Methionine 1 µl), then kept on 30 °C for 90 min. Next, the in vitro proteolysis of BMAL1 was carried out by incubating 5 μl BMAL1 substrate with 0.25 μg of 20S proteasome (Boston Biochem) and 2 μg of purified REGγ in in vitro degradation buffer (10 mM Tris-HCl pH: 7.5, 10 mM KCl, 10% glycerol) for 3–5 h in 25 μl reaction volume at 30 °C with suitable measures. The aliquots of the reaction were finally utilized for western blot analysis.

### Gel base and real-time qPCR

Total RNA was extracted from cells and brain SCN sections in RNA isolation reagent Trizol (Takara). cDNA was synthesized using 2 μg of whole-cell RNA in a total 20 μl reaction system with 5× RT SuperMix (Vazyme, China) and RNase-free ddH_2_O. The reverse transcribed RNA was then used for gel base and real-time qPCR analysis.

For gel base analysis, the following reaction mix in final volume of 20 μl was used: forward and reverse primer mix 2 μl, 10× Taq Buffer (Mg^2+^ plus) 10 μl, cDNA template 2 μl, and mQ water 6 μl. And then run on PCR instrument with the following program: step1 95 °C 5 min, step2 for 30 cycles, (95°C 30 s, 58 °C 30 s, 72 °C 45 s), step3 72°C 10 min, step4 16 °C ~.

For real-time qPCR analysis, aliquots of the cDNA were prepared in total volume of 20 μl reaction including 10 μl SYBR Green (Vazyme, China), 0.8 μl of primers mix, 0.8 μl cDNA, and 8.4 μl mQ water and then executed on Quantstudio PCR using the following procedure: step1, 95 °C for 10 min followed by step2 40 cycles at 95 °C for 30 s, 58 °C for 30 s, 72 for 45 s, and step 3, 72 °C for 10 min. The gene-specific primers for mRNA analysis are given in Supplementary Table 1.

### Cycloheximide assay and doxycycline

For cycloheximide assay, primary MEF WT/KO and SY5Y ShN/ShR cells were treated with 10 μl cycloheximide in DMEM having 10% FBS and 1% PS at the indicated time points and then collected the cells for western blot analysis. REGγ-inducible 293 cells were treated with doxycycline to induce REGγ followed by cycloheximide assay.

### Statistical analysis

Statistical analyses were performed using GraphPad Version 6.0, Prism Software Inc., San Diego, CA. Med Associates Running Wheel Data Analysis Software was used to compare the circadian time periods. All the data were expressed as mean ± standard deviation (SD). Differences between two groups or more were analyzed using Student’s *t* test. *p* values of <0.05 (*p* < 0.05*) were considered to be of statistical significance.

## Supplementary information

Supplementary Figure Legends

Figure S1. Light shock in early night induce circadian phase delay in REGγ KO mice.

Figure S2. REGγ deficiency up regulates circadian genes expression in SCN of REGγ KO mice.

Figure S3. REGγ deficiency upregulates circadian clock specific genes in MEF KO and SY5Y ShR cells.

Figure S4. Inhibition of REGγ-GSK3β signaling increases the expression of circadian genes in MEF WT and SY5Y ShR cells.

Table S1

## Data Availability

Data are available on request from the authors.
