# Supplementary material for: REGγ regulates circadian clock by modulating BMAL1 protein stability
Source: Cell Death Discov. 2021 Nov 5;7:335. doi: 10.1038/s41420-021-00704-9 (PMC8571338; doi:10.1038/s41420-021-00704-9)
Supplement: Supplementary file 1 — Supplementary Figure Legends [file 41420_2021_704_MOESM1_ESM.docx]

**Supplementary Figure Legends:**

**Figure S1. Light shock in early night induce circadian phase delay in REGγ KO mice.** Actogram of (A) REGγ WT and (B) REGγ KO mice entrained in complete dark/dark (DD) for 7 days and then conferred a light shock for 15 minutes on day 8 in dark/dark at CT15 early night. Red star and black arrow show the day when light shock was provided.

**Figure S2. REGγ deficiency upregulates circadian genes expression in REGγ KO mice SCN.** (A) Real-time qPCR analysis of circadian genes *Per1*, *Per2*, *Cry1*, *Clock*, *Bmal1*, *Rorα* and *REGγ* mRNA expression in SCN of REGγ WT and KO mice. (B) Gel base PCR analysis of circadian genes *Per1*, *Per2*, *Cry1*, *Clock*, *Bmal1*, *Rorα* and *REGγ* in REGγ WT and KO mice SCN. The data for real-time qPCR represent the mean ± SEM. ***p < 0.001, **p < 0.01, *p < 0.05; t test, SCN WT vs. KO.

**Figure S3. REGγ deficiency upregulates circadian clock specific genes in MEF KO and SY5Y ShR cells.** (A, B) Real-time qPCR and gel base PCR analysis of circadian genes *Per1*, *Per2*, *Cry1*, *Clock*, *Bmal1*, *Rorα* and *REGγ* in primary MEF WT and KO cells collected from REGγ HZ pregnant mice WT and KO embryos. (C, D) Real-time qPCR and gel base PCR analysis of circadian genes *Per1*, *Per2*, *Cry1*, *Clock*, *Bmal1*, *Rorα* and *REGγ* in stable knockdown SY5Y ShN and ShR cells. Error bars for Real time-qPCR represent mean ± SEM. ***p < 0.001, **p < 0.01, *p < 0.05; t test, WT vs. KO. ShN vs. ShR.

**Figure S4. Inhibition of REGγ-GSK3β signaling increases the expression of circadian genes in MEF WT and SY5Y ShR cells.** Gel base PCR analysis of circadian genes *Per1*, *Per2*, *Cry1*, *Clock* and *Bmal1* in REGγ WT/KO MEF and stable knockdown SY5Y ShN/ShR cells. (A, B) represent the circadian genes in normal condition in MEF and SY5Y cells following the 50% serum shock time course experiment. (C, D) MEF and SY5Y cells were treated with GSK-3β inhibitor (S1263, 10μM) followed by the 50% serum shock and then collected the cells for mRNA analysis. Result showed that inhibition of GSK-3β causes the upregulation of circadian clock specific genes in REGγ KO MEF and REGγ knockdown SY5Y ShR cells followed by perturbed circadian pattern.
