# Supplementary figures and images for: REGγ regulates circadian clock by modulating BMAL1 protein stability
Source: Cell Death Discov. 2021 Nov 5;7:335. doi: 10.1038/s41420-021-00704-9 (PMC8571338; doi:10.1038/s41420-021-00704-9)

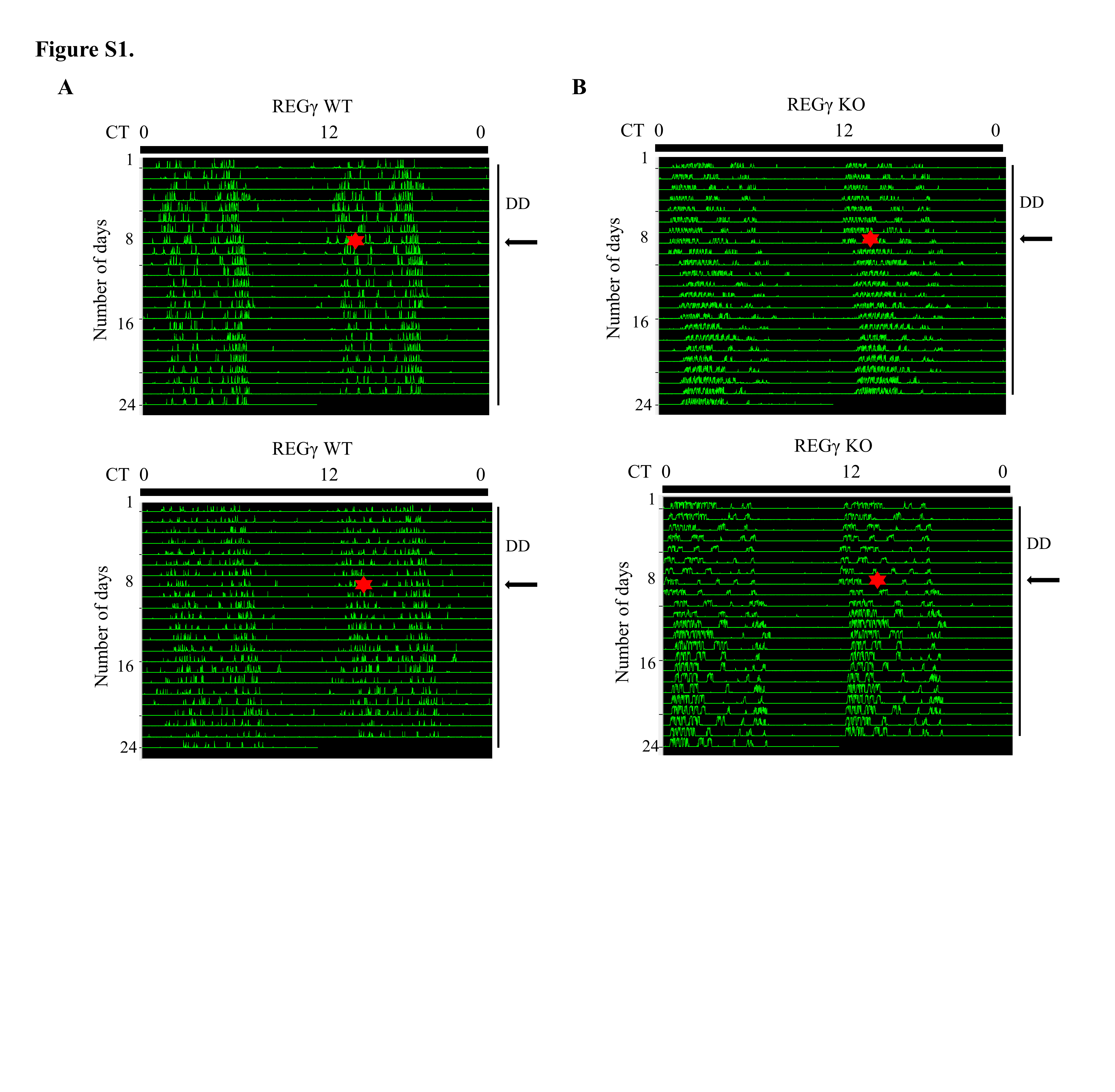

Supplement: Supplementary file 2 — Figure S1. Light shock in early night induce circadian phase delay in REGγ KO mice. [file 41420_2021_704_MOESM2_ESM.tif]

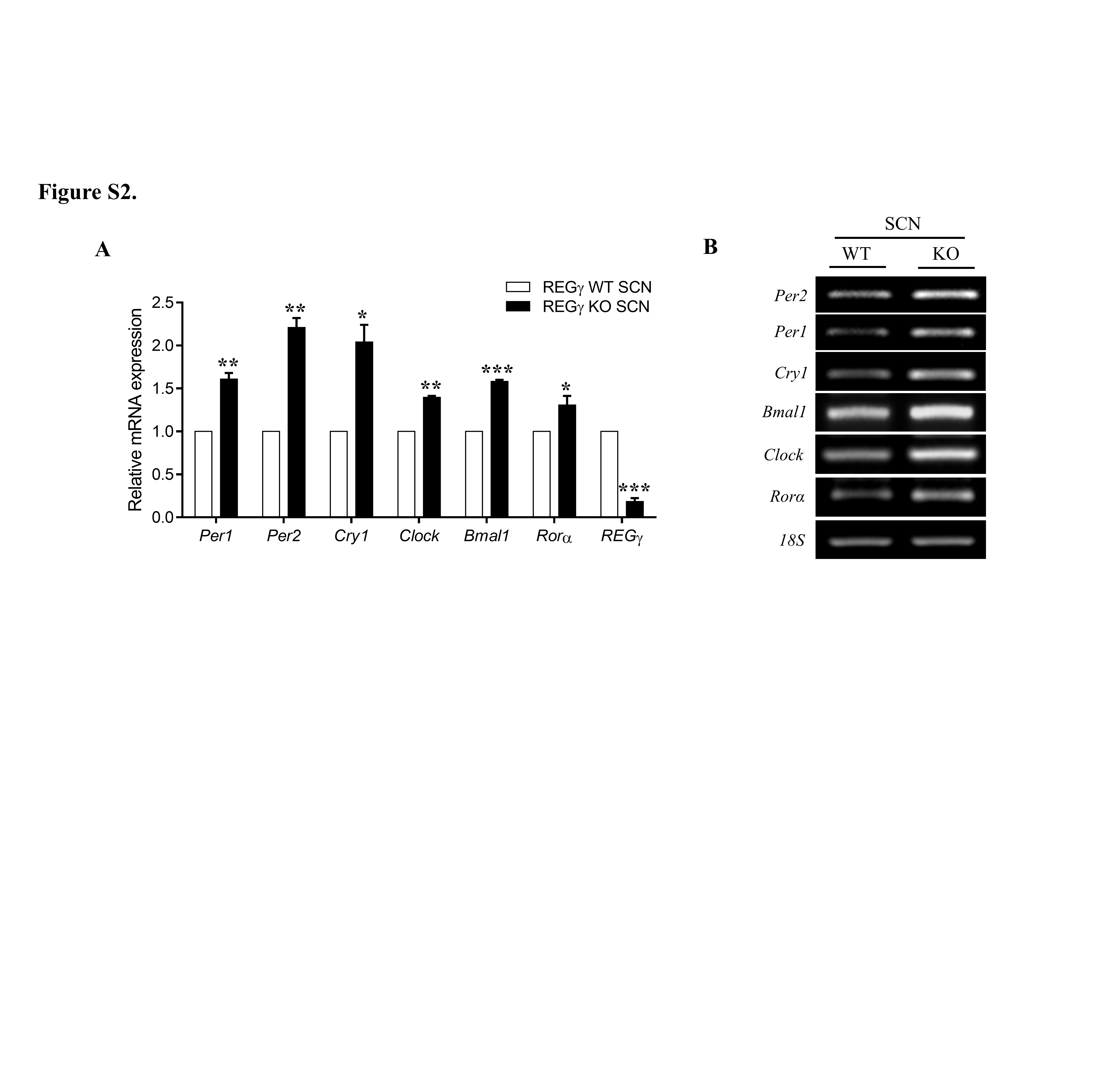

Supplement: Supplementary file 3 — Figure S2. REGγ deficiency up regulates circadian genes expression in SCN of REGγ KO mice. [file 41420_2021_704_MOESM3_ESM.tif]

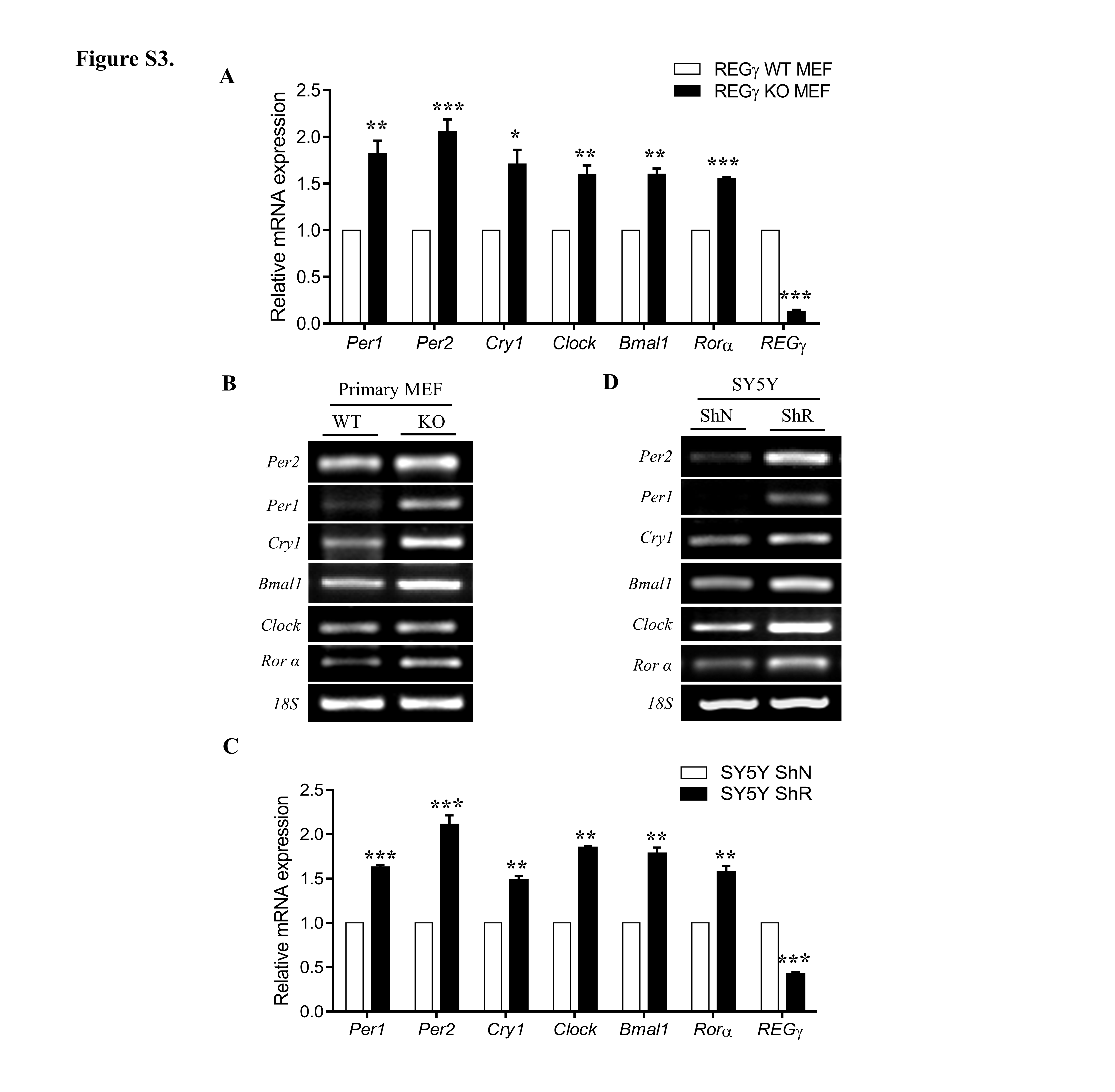

Supplement: Supplementary file 4 — Figure S3. REGγ deficiency upregulates circadian clock specific genes in MEF KO and SY5Y ShR cells. [file 41420_2021_704_MOESM4_ESM.tif]

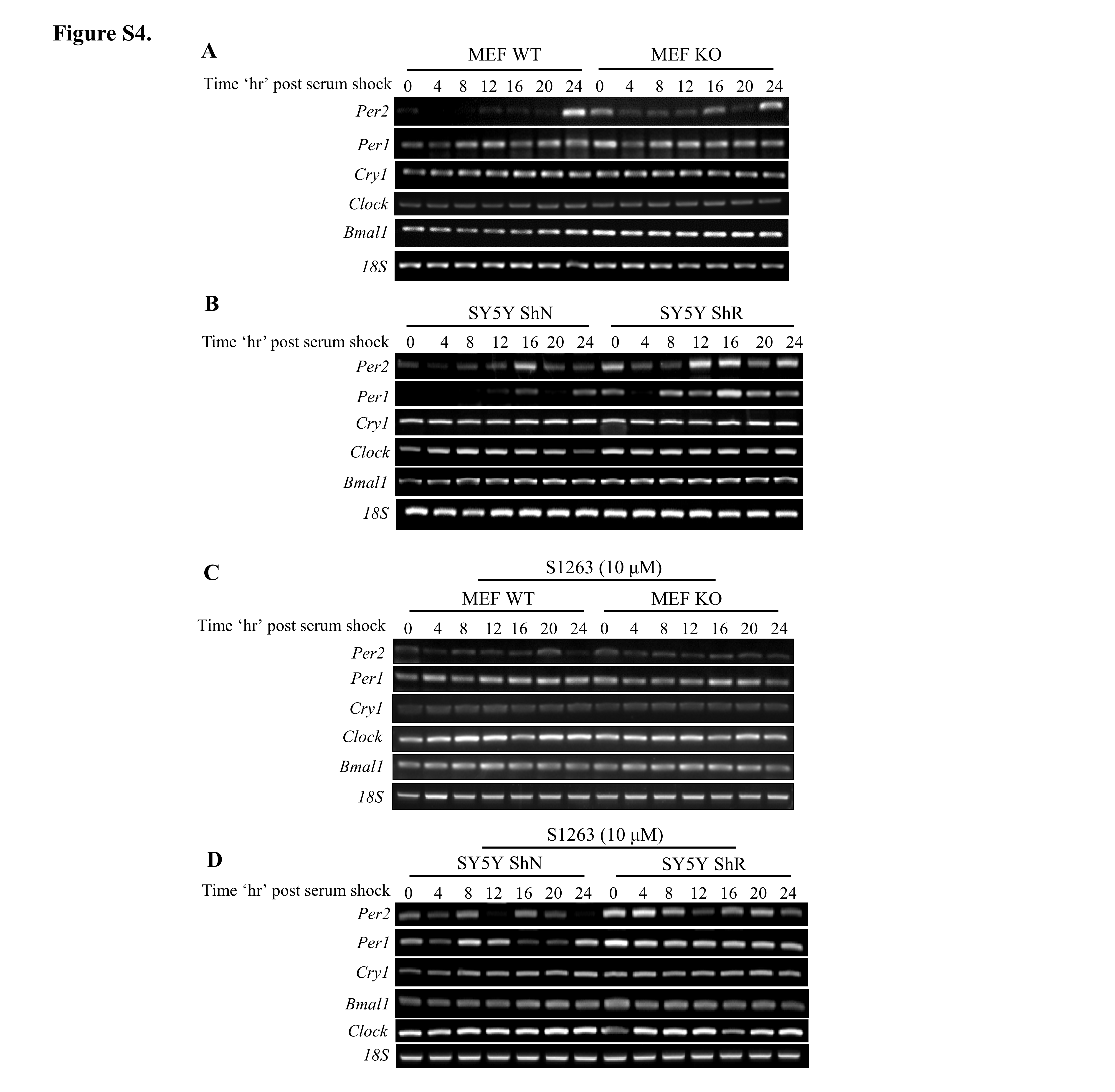

Supplement: Supplementary file 5 — Figure S4. Inhibition of REGγ-GSK3β signaling increases the expression of circadian genes in MEF WT and SY5Y ShR cells. [file 41420_2021_704_MOESM5_ESM.tif]
