# Supplementary material for: REGγ regulates circadian clock by modulating BMAL1 protein stability
Source: Cell Death Discov. 2021 Nov 5;7:335. doi: 10.1038/s41420-021-00704-9 (PMC8571338; doi:10.1038/s41420-021-00704-9)
Supplement: Supplementary file 6 — Table S1 [file 41420_2021_704_MOESM6_ESM.docx]

**Supplementary Material**

**Table S1. The specific primer sequences for PCR analysis.**

| **Gene** | **Primer sequence forward 5ʹ-3’** | **Primer sequence reverse 5ʹ-3’** |  |
| --- | --- | --- | --- |
| *Per1* (Mus) | GATGTGGGTGTCTTCTATGGC | AGGACCTCCTCTGATTCGGC |  |
| *Per1* (Homo) | GCCAACCAGGAATACTACCAGC | GTGTGTACTCAGACGTGATGTG |  |
| *Per2* (Mus) | AAAGCTGACGCACACAAAGAA | ACTCCTCATTAGCCTTCACCT |  |
| *Per2* (Homo) | CTTCAGCGATGCCAAGTTTGT | CGGATTTCATTCTCGTGGCTTT |  |
| *Cry1* (Mus) | CACTGGTTCCGAAAGGGACTC | CTGAAGCAAAAATCGCCACCT | |
| *Cry1* (Homo) | CTCCTCCAATGTGGGCATCAA | CCACGAATCACAAACAGACGG |  |
| *Clock* (Mus) | ATGGTGTTTACCGTAAGCTGTAG | CTCGCGTTACCAGGAAGCAT |  |
| *Clock* (Homo) | TGCGAGGAACAATAGACCCAA | ATGGCCTATGTGTGCGTTGTA |  |
| *Bmal1* (Mus) | ACAGTCAGATTGAAAAGAGGCG | GCCATCCTTAGCACGGTGAG |  |
| *Bmal1* (Homo) | AAGGGAAGCTCACAGTCAGAT | GGACATTGCGTTGCATGTTGG |  |
| *18S* (Mus) | GGACACGGACAGGATTGACA | GACATCTAAGGGCATCACAG |  |
| *REGγ* | ACAAGTGAGGCAGAAGAC | ATCATGGCTATTGGTGAG |  |
| *Rorα* (Homo) | ATCTCCGACTCCTTACCTTCG | AGGGGCTCCTACACTTTACAG |  |
| *Rorα* (Mus) | GTGGAGACAAATCGTCAGGAAT | TGGTCCGATCAATCAAACAGTTC |  |
